# Disruption of SUMO-Specific Protease 2 Induces Mitochondria Mediated Neurodegeneration

**DOI:** 10.1371/journal.pgen.1004579

**Published:** 2014-10-09

**Authors:** Jiang Fu, H.-M. Ivy Yu, Shang-Yi Chiu, Anthony J. Mirando, Eri O. Maruyama, Jr-Gang Cheng, Wei Hsu

**Affiliations:** 1 Department of Biomedical Genetics, Center for Oral Biology, Stem Cell and Regenerative Medicine Institute, University of Rochester Medical Center, Rochester, New York, United States of America; 2 UNC-Neuroscience Center, University of North Carolina at Chapel Hill, Chapel Hill, North Carolina, United States of America; 3 Wilmot Cancer Institute, University of Rochester Medical Center, Rochester, New York, United States of America; University of Minnesota, United States of America

## Abstract

Post-translational modification of proteins by small ubiquitin-related modifier (SUMO) is reversible and highly evolutionarily conserved from yeasts to humans. Unlike ubiquitination with a well-established role in protein degradation, sumoylation may alter protein function, activity, stability and subcellular localization. Members of SUMO-specific protease (SENP) family, capable of SUMO removal, are involved in the reversed conjugation process. Although SUMO-specific proteases are known to reverse sumoylation in many well-defined systems, their importance in mammalian development and pathogenesis remains largely elusive. In patients with neurodegenerative diseases, aberrant accumulation of SUMO-conjugated proteins has been widely described. Several aggregation-prone proteins modulated by SUMO have been implicated in neurodegeneration, but there is no evidence supporting a direct involvement of SUMO modification enzymes in human diseases. Here we show that mice with neural-specific disruption of SENP2 develop movement difficulties which ultimately results in paralysis. The disruption induces neurodegeneration where mitochondrial dynamics is dysregulated. SENP2 regulates Drp1 sumoylation and stability critical for mitochondrial morphogenesis in an isoform-specific manner. Although dispensable for development of neural cell types, this regulatory mechanism is necessary for their survival. Our findings provide a causal link of SUMO modification enzymes to apoptosis of neural cells, suggesting a new pathogenic mechanism for neurodegeneration. Exploring the protective effect of SENP2 on neuronal cell death may uncover important preventive and therapeutic strategies for neurodegenerative diseases.

## Author Summary

Protein modification by SUMO is a reversible and evolutionarily conserved process. Members of the SUMO-specific protease (SENP) family are known to reverse SUMO-conjugation in many defined systems, but their importance in mammalian development and pathogenesis remains largely elusive. Although SUMO-conjugated proteins have been shown to aberrantly accumulate in patients with neurodegeneration, there is no evidence supporting a direct involvement of SUMO modification enzymes in human diseases. This study reveals that disruption of SENP2 causes neurodegeneration through modulation of mitochondrial morphogenesis. Our findings provide a causal link of SUMO modification enzymes to cell survival, suggesting a new pathogenic mechanism for neurodegeneration. Exploring the protective effect of SENP2 on neuronal cell death may uncover important preventive and therapeutic strategies for neurodegenerative diseases.

## Introduction

Emerging evidence suggests the importance of protein modification by Small Ubiquitin-related Modifier (SUMO) in neural development and function [1]–[3]. Abnormal SUMO modification has been found in several neurodegenerative diseases, characterized by progressive loss or dysfunction of neurons [4]–[6]. Unlike ubiquitin with a well-established role in protein degradation, SUMO is involved in protein trafficking, cell proliferation and survival, as well as ubiquitin-mediated proteolysis [7]–[11]. Covalent conjugation of SUMO to protein substrates, also known as sumoylation, is a reversible process catalyzed by SUMO ligases [12],[13]. The removal of SUMO, also known as desumoylation, is mediated by SUMO proteases [14], [15]. Although these proteins have been shown to reverse sumoylation in various physiological systems, their roles in mammalian development and disease remain largely unknown. SUMO-specific protease 2 (SENP2) is found in three alternatively spliced forms exhibiting differential subcellular localizations [16]. Genetic inactivation of *Senp2* reveals its requirement in development of trophoblast stem cell niches and lineages during development of the placenta [17]. Although SENP2 mutants display embryonic defects including brain and heart abnormalities, they are likely associated with placental insufficiency which requires further investigation [17], [18].

Enhanced sumoylation and accumulation of SUMO-conjugated proteins have been widely observed in patients with various neurodegenerative disorders [19]–[22]. Among the most notable ones are polyglutamine disorders, including Huntington's disease (HD) caused by a trinucleotide expansion, and neuronal intranuclear inclusion disease (NIID). The encoded CAG expansions result in production of toxic proteins carrying extended glutamine repeats. In HD, SUMO1 conjugation of the disease protein Huntingtin (Htt) contributes to the disease pathology possibly by stabilizing the toxic Htt [20]. SUMO-modified targets/substrates also accumulate in the nuclear aggregates of NIID, a multisystem neurodegenerative disease characterized by large intranuclear inclusions in neurons of the central and peripheral nervous systems [21]. In autosomal recessive juvenile parkinsonism, the SUMO pathway might affect protein degradation mediated by the disease protein Parkin, an E3-ubiquitin ligase [23]. Targeting the SUMO pathway may offer new strategies for disease prevention and therapy. However, there is no evidence indicating a direct involvement of SUMO modification regulators/enzymes in neurodegenerative disease. Information providing a causal link of SUMO dysregulation to neural cell survival is also very limited.

## Results

### SENP2 deficiency causes neurodegeneration

**Figure 1:**
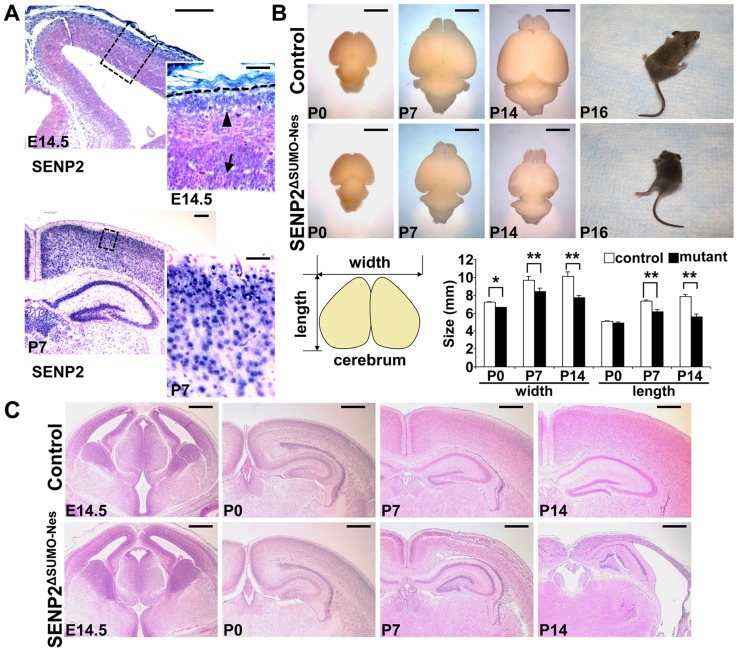
Disruption of SENP2 in the neural progenitors causes neurodegeneration. (A) In situ hybridization analysis shows the expression of SENP2 in the neural progenitors and differentiated cells in the mouse cerebral cortex at embryonic day 14.5 (E14.5), postnatal day 7 (P7). Arrow, arrowhead and broken line indicate neural progenitors in the subventricular zone, differentiated cells in the cortical plate and outer edge of the cerebral cortex, respectively. Enlargement of the insets are shown at right. (B) Images show gross morphology of the control and SENP2^ΔSUMO-Nes^ brains at P0, P7 and P14, and development of paralysis in the SENP2 mutant (100%, n = 20). Graph illustrates reduction of the brain size caused by the SENP2 deletion (*, p<0.05; **, p<0.01, n = 3). (C) Hematoxylin and eosin staining analyzes development of the control and SENP2^ΔSUMO-Nes^ brains at E14.5, P0, P7 and P14. The genotype for control mice is Nestin-Cre; SENP2^SUMO^Fx/+ or SENP2^SUMO^Fx/Fx. Scale bars, 250 µm (A, left panel); 50 µm (A, right panel); 3 mm (B); 500 µm (C).

We previously created a mouse strain carrying a null allele of SENP2 [17]. The knockout of SENP2 led to severe developmental abnormalities in trophoblast stem cell niches and lineages during placentation [17]. Although brain and heart deformities were also detected in the SENP2-null embryos (Figure S1, Maruyama et al., unpublished, and [18]), we speculated these are secondary defects due to placental insufficiencies [17]. To analyze the involvement of SENP2 and the importance of SUMO modification in neural development and disease, we first examined its expression pattern. In situ hybridization detected the presence of SENP2 mRNA in the developing mouse brain at embryonic day 14.5 (E14.5) and postnatal day 0 (P0), P7 and P14 (Figure 1A). SENP2 was expressed in subventricular neural progenitors and differentiated cells of the cerebral cortex (Figure 1A). To definitively assess our speculation on the contribution of placental deficiencies to the embryonic deformities, we took a genetic approach by creating a mouse model deficient for SENP2 during neural development. A new mouse strain carrying a *SENP2^ΔSUMO^Fx* allele, permitting removal of the protease core domain using the Cre-loxP system, was generated (Figure S2). The presence of Cre caused an in-frame deletion, resulting in production of a SENP2 mutant deficient for the SUMO protease activity. Using EIIa-Cre to remove the protease core domain, we generated a mouse strain carrying *SENP2^ΔSUMO^* mutant allele expressing the truncated SENP2 (Figure S3). The *SENP2^ΔSUMO^Δ/Δ* embryos were significantly smaller or underdeveloped compared to their *SENP2^ΔSUMO^+/+* and *SENP2^ΔSUMO^+/Δ* littermates at E10.5 (Figure S3A–B). Development of all three trophoblast layers was severely impaired in the homozygous mutants (Figure S3C–J). These extraembryonic and embryonic defects are highly reminiscent to the SENP2 nulls [17], suggesting that the protease core domain deletion results in a loss of function mutation. We also were able to obtain mice heterozygous for the deleted allele without any noticeable abnormality, further suggesting that there is no dominant phenotype associated with the mutation.

Next, we generated a SENP2^ΔSUMO-Nes^ model, in which SENP2 is ablated in the neural progenitor cells by Nestin-Cre (Figure S4). At newborn, no obvious defects associated with the deletion could be detected, including neuronal differentiation (Fu and Hsu, unpublished), indicating that SENP2 is not essential for embryonic neural development. The embryonic deformities observed in the SENP2 nulls were attributed to placental insufficiency. However, the SENP2^ΔSUMO-Nes^ mice displayed movement difficulties at P10. They developed paralysis around P16 (Figure 1B and Supplementary Video S1; 100% penetrance, n = 20 SENP2^ΔSUMO-Nes^ mutants collected from 10 litters), and died at the age of 3 weeks. The size of the mutant brains was slightly smaller comparable to the control at P0, but later on exhibited a gradual reduction (Figure 1B). At P14, the mutant brain looked transparent, and was much smaller than the control (Figure 1B; *, p<0.05; **, p<0.01, n = 3). Histology revealed no obvious defects at P0 but severe brain abnormalities at P7 and P14 associated with the SENP2 deficiency (Figure 1C). The cerebral cortex of SENP2^ΔSUMO-Nes^ became significantly thinner and malformed. Other CNS regions, e.g. midbrain, cerebellum, hippocampus and spinal cord were also affected by the mutation although the phenotypes were less severe (Figure S5). The results suggested an essential role of SENP2 in neural development at postnatal, but not prenatal, stages.

### SENP2 is essential for protection of mitochondria-dependent apoptosis

**Figure 2:**
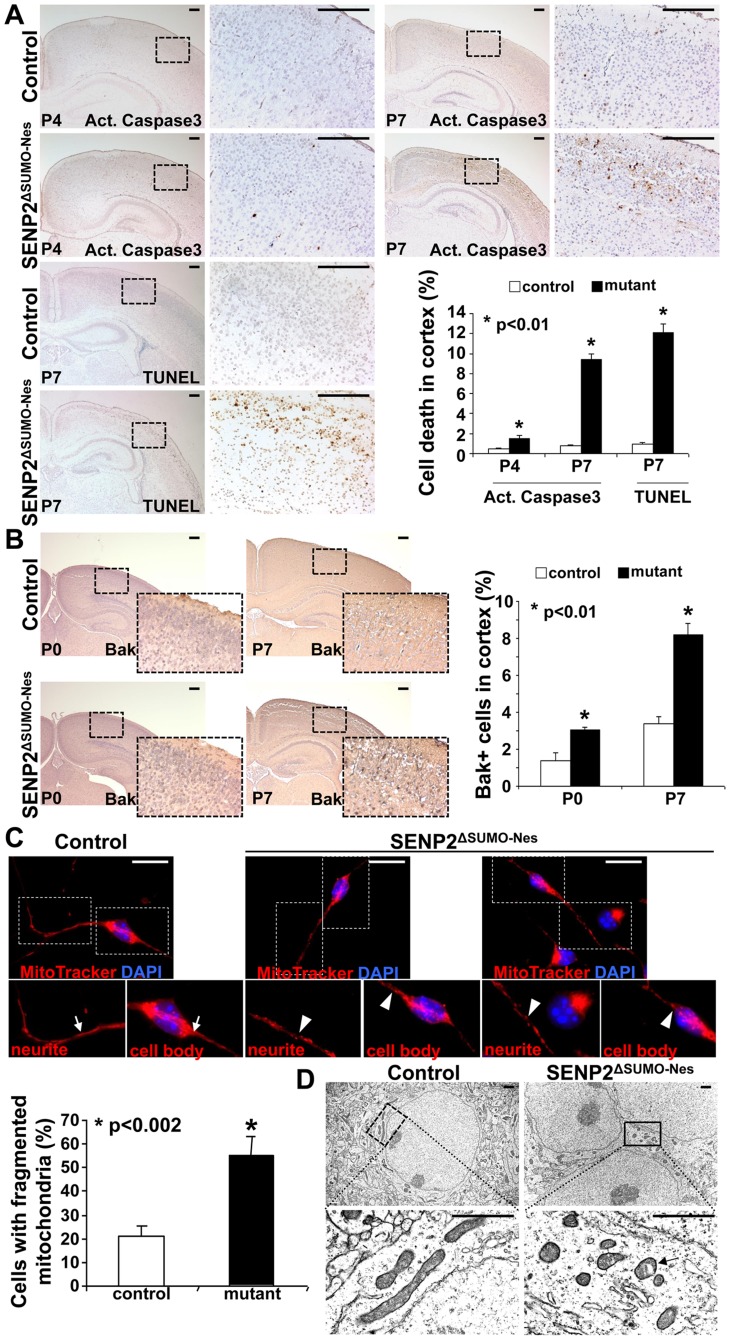
Neuronal cell death caused by the loss of SENP2 is associated with dysregulation of mitochondrial dynamics. (A) Immunostaining of active (Act.) Caspase3 and TUNEL staining examine apoptotic cells in the P4 and P7 cerebral cortex of control and SENP2^ΔSUMO-Nes^. Graph shows percentages of the positively stained cells (*, p<0.01, n = 3, mean ± SEM). (B) Immunostaining of the N-terminus and BH-1 domain of the pro-apoptotic protein Bak, whose exposure indicates its activation, examines the mitochondria-dependent apoptosis in the cerebral cortex of control (genotype: Nestin-Cre; SENP2^SUMO^Fx/+ and SENP2^SUMO^Fx/Fx) and SENP2^ΔSUMO-Nes^ at P0 and P7. Graph shows percentages of the Bak positive cells (*, p<0.01, n = 3, mean ± SEM). (C) Mitochondrial dynamics of the neurons derived from the P7 cerebral cortex of control and SENP2^ΔSUMO-Nes^ are analyzed by MitoTracker labeling. Arrows and arrowheads indicate tubular/rod-like and fragmented mitochondria, respectively. Graph shows percentages of cells containing fragmented mitochondria (*, p<0.002, n = 3, mean ± SEM). (D) Sections of the P7 control and SENP2^ΔSUMO-Nes^ cerebral cortex are examined by electron microscopy. Arrow indicates alterations of the mitochondria inner membrane. Scale bars, 200 µm (A–B); 20 µm (C); 1 µm (D).

The neurodegenerative phenotype of SENP2^ΔSUMO-Nes^ prompted us to examine programmed cell death affected by the mutation. Immunostaining of active Caspase 3 and TUNEL analysis revealed that abnormal apoptosis is, not detectable at P0, but increased at P4 (Caspase 3: 0.46±0.12% in control vs. 1.52±0.33% in mutant) and highly enhanced at P7 (Caspase 3: 0.82±0.08% in control vs. 9.4±0.59% in mutant; TUNEL: 0.91±0.17% in control vs. 12.09±0.87% in mutant) (Figure 2A, p<0.01, ∼700 cells were counted in each of 3 independent experiments, mean ± SEM). The apoptotic abnormality, albeit less severe at this stage, was also observed in other CNS regions (Figure S6). To further elucidate the mechanism underlying the neural cell death of SENP2^ΔSUMO-Nes^, we examined expression of the activated form of Bak, a proapoptotic effector which promotes programmed cell death through modulation of mitochondrial morphogenesis [24], . In the SENP2^ΔSUMO-Nes^ cerebral cortices, Bak activation is stimulated at P0 (1.39±0.41% in control vs. 3.03±0.17% in mutant) and P7 (3.4±0.36% in control vs. 8.21±0.59% in mutant), suggesting an association of mitochondrial dysfunction with the SENP2 mutation (Figure 2B, p<0.01, ∼700 cells were counted in each of 3 independent experiments, mean ± SEM). Neurons derived from the cerebral cortices of mouse embryonic brains were then cultured in vitro for examination of mitochondrial dynamics. Fluorescent labeling of the mitochondria revealed a more than 2.5-fold increase of neurons containing fragmented, but not tubular/rod-like, mitochondria in the cell body and neurite caused by the mutation (20.8±4.4% in control vs. 55.3±7.8% in mutant) (Figure 2C, p<0.002, ∼200 neurons were counted in each of 3 independent experiments, mean ± SEM). Electron microscopy analysis of the P7 brain sections further identified fragmentation of the mitochondria in the cerebral cortical neurons of SENP2^ΔSUMO-Nes^ (Figure 2D). The mitochondrial cisternae are generally intact although few of them show alterations on the inner membrane. The results thus suggested a protective effect of SENP2 on neuronal cell survival. SENP2 plays an essential role in the regulation of mitochondrial dynamics during postnatal development of CNS.

**Figure 3:**
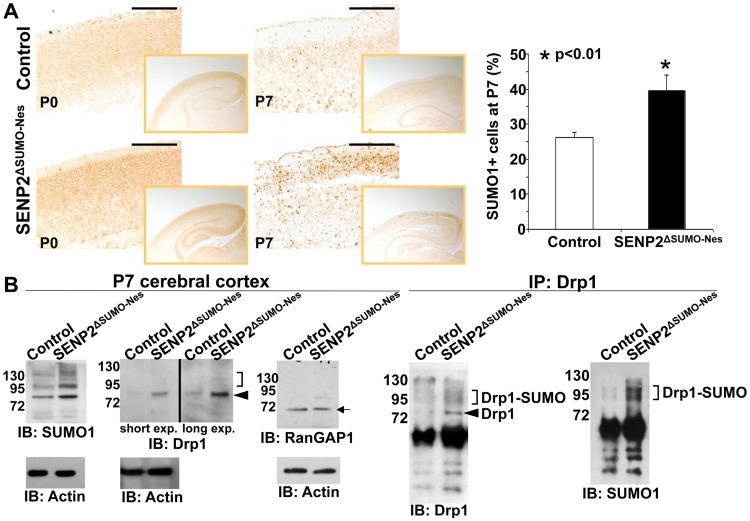
SENP2 deficiency enhances Drp1 sumoylation and stabilization in the developing cerebral cortex. (A) The overall levels of sumoylation in the P0 and P7 control (genotype: Nestin-Cre; SENP2^SUMO^Fx/+ and SENP2^SUMO^Fx/Fx) and SENP2^ΔSUMO-Nes^ brains are assessed by immunostaining of SUMO1. Graph illustrates percentages of the SUMO1 positive cells at P7 (*, p<0.01, n = 3, mean ± SEM). (B) Immunoblot (left) and immunoprecipitation-immunoblot (right) analyses of the P7 control and SENP2^ΔSUMO-Nes^ brains examines the SUMO modification (brackets), and protein stability of Drp1 (arrowheads) and RanGAP1 (arrow) affected by the SENP2 mutation. Data shown here are representatives of three independent experiments. Scale bars, 200 µm (A).

We then examined whether the SENP2 deficiency causes imbalances of sumoylation, resulting in accumulations of SUMO-conjugated proteins. Immunostaining of SUMO1 showed increased levels of the sumoylated proteins (26.1±1.5% in control vs. 39.4±4.5% in mutant), indicating that SENP2 deficiency induces hyper-sumoylation (Figure 3A, p<0.01, ∼700 cells were counted in each of 3 independent experiments, mean ± SEM). Although SENP2 was shown to regulate the Mdm2-p53 pathway [16], [17], the expression and the activity of p53 and Mdm2 were not altered in these mutants (Fu and Hsu, unpublished). The neural defects caused by the SENP2 deletion most likely were not associated with p53-induced apoptosis, which is a mitochondrial independent event. Examination of protein extracts isolated from the P7 cerebral cortices revealed an elevation of SUMO1 association in the mutants (Figure 3B). The loss of SENP2 activated Bak (Figure 2B), which has been shown to promote sumoylation of Dynamin regulated protein 1 (Drp1) and its association with mitochondria during programmed cell death [24], [25]. Therefore, we tested if Drp1 is affected in the SENP2^ΔSUMO-Nes^ mutants. Not only the stability (1.9-fold), but also SUMO1 association with Drp1 (2.7-fold), was enhanced by the mutation while RanGAP1, a known substrate of SENP2, did not appear to be affected (Figure 3B). We then examined the mitochondrial association of Drp1 in primary neurons derived from the cerebral cortices of mouse embryonic brains. The mutation apparently promoted Drp1 association with the mitochondria (Fig. S7). The results implied that dysregulation of Drp1 may cause mitochondrial defects, leading to the development of neurodegeneration in the SENP2^ΔSUMO-Nes^ mutants.

### SENP2 in SUMO-mediated regulation of Drp1

**Figure 4:**
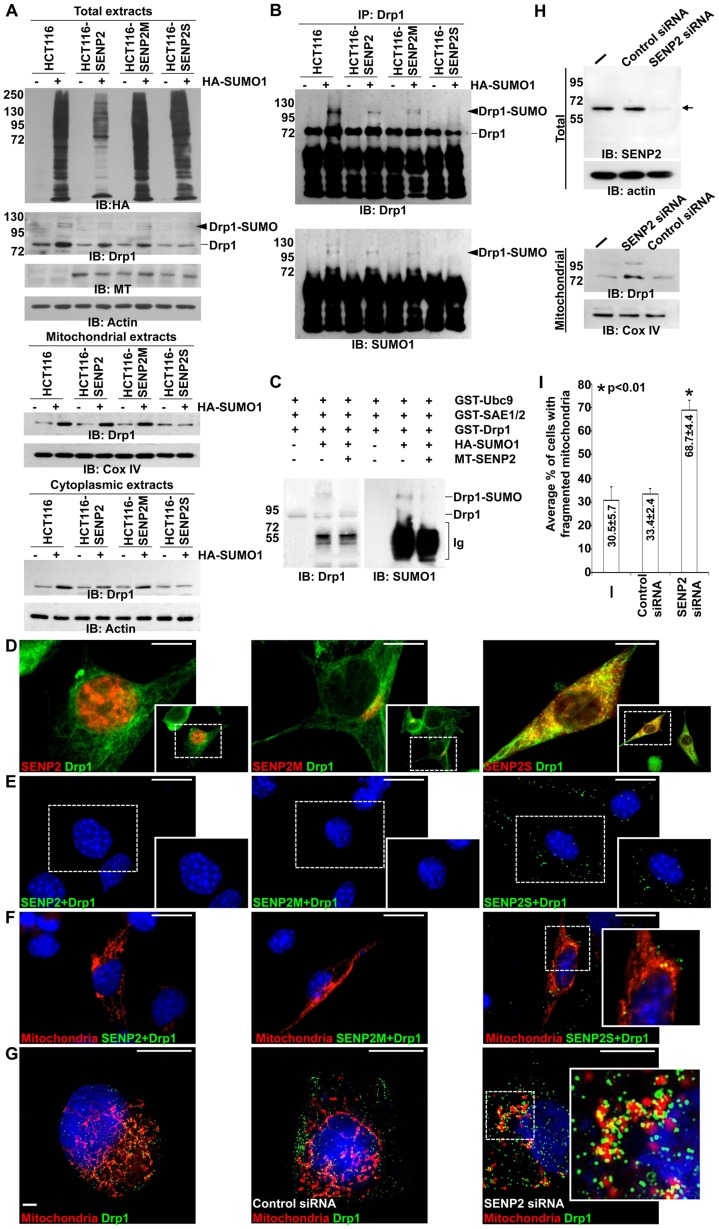
SUMO1 modification of Drp1 is regulated by SENP2. (A) Immunoblot (IB) analysis examines total cell expression of HA-SUMO1, Drp1, myc tagged (MT)-SENP2 isoforms and Actin, as well as the mitochondrial and cytoplasmic expression of Drp1, and Cox IV or Actin, in HCT116 cells and in HCT116 stably transformed variants, HCT116-SENP2, HCT116-SENP2M and HCT116-SENP2S. Overexpression of HA-SUMO1 induces sumoylation in the total fraction of HCT116 cells and in HCT116 stably transformed variants. The level of Drp1 is only increased in HCT116, HCT116-SENP2 and HCT116-SENP2M, but not HCT116-SENP2S cells. The level of Actin and Cox IV, a mitochondrial marker, are used as protein loading controls. (B) Immunoprecipitation (IP) followed by IB analyzes the desumoylation of Drp1 by SENP2 isoforms in HCT116 and HCT116 stably transformed variants. The SUMO-associated Drp1 is only absent in HCT116-SENP2S, but not HCT116, HCT116-SENP2 and HCT116-SENP2M. (C) In vitro reconstitution assay using recombinant enzymes and substrates reveals that SUMO conjugation of Drp1 is reversed by purified SENP2. Drp1 is sumoylated by recombinant Ubc9, SEA1/2 and SUMO1 proteins, followed by desumoylation with purified SENP2. (D) Co-immunostaining of endogenous Drp1 and transiently expressed myc tagged SENP2 isoforms examines their subcellular localizations. Images are enlargements of the insets shown at right. (E) Association of endogenous Drp1 and different SENP2 isoforms is determined by proximity ligation assay using a Duolink system detecting in-cell protein interaction. Dotted staining represents the association of Drp1 with SENP2-S, but not SENP2 and SENP2-M in C3H10T1/2 cells. (F) Double labeling with the proximity ligation assay and the DsRed2-Mito staining detects the mitochondrial interaction of Drp1 and SENP2S. (G) Double labeling of DsRed2-Mito and Drp1 reveals its accumulations to the mitochondria upon the knockdown of SENP2. Enlargements of the insets are shown at right (E–G). (H) IB analysis shows effectiveness of the SENP2 knockdown by siRNA, and mitochondrial stabilization and sumoylation of Drp1 caused by the SENP2 reduction. The levels of actin and Cox IV are analyzed as loading controls. (I) Graph illustrates the statistical analysis of mitochondrial morphology (p<0.01, n = 3, mean ± SEM). Scale bars, 20 µm (D); 50 µm (E, F); 20 µm (G).

Drp1 has been implicated in neural degenerative diseases with disruption of mitochondrial dynamics [26], [27]. To test if Drp1 plays a role in this pathogenic process, we investigated its regulation by SENP2. Our previous report showed that three gene products of *SENP2* (SENP2, SENP2M and SENP2S), generated by alternative splicing, leading to the use of distinct translation initiation sites, exhibit distinct subcellular localizations and functions [16]. The SENP2, SENP2M and SENP2S isoforms are predominately located to the nucleus, cytoplasmic vesicles and perinuclear region, and cytoplasm, respectively [16]. First, we examined which of these isoforms might be involved in the regulation of Drp1 using a parental cell line and its stably transformed variants, which express high levels of different isoforms [16]. Whole cells or mitochondria only prepared from these cell lines were used to isolate extracts, followed by protein analysis. Overexpression of a HA tagged SUMO1 led to hyper-sumoylation of total as well as the mitochondrial proteins in the parental cells which occurs less effectively in all SENP2 variants (Figure 4A). SUMO1 also promotes total cell, cytoplasmic and mitochondrial accumulation of Drp1, suggesting that its stability is modulated by sumoylation. However, this regulatory process, not affected by SUMO2 and SUMO3, is apparently a SUMO1-specific regulation (Figure S8A, B). Moreover, high levels of SENP2S, but not SENP2 and SENP2M, prevented the SUMO1-induced accumulation of Drp1 to the mitochondria (Figure 4A). SENP2S also decreased the SUMO1-induced accumulation of Drp1 in the cytoplasm. Thus suggests that the Drp1 reduction mediated by SENP2S is caused by protein degradation rather than decreased targeting to the mitochondria (Figure 4A). Immunoprecipitation-immunoblot analysis further showed that the SUMO1-association of endogenous Drp1 is eliminated by SENP2S, but not other isoforms (Figure 4B). Although certain levels of reduction were detected in the SENP2 and SENP2M analyses, these might be attributed to the disruption of cellular compartmentalization in vitro. To further examine the ability of SENP2 to remove SUMO1 from Drp1, we used in vitro reconstitution analysis (Figure 4C). Recombinant enzymes, including Ubc9 and SAE1/2, were first utilized to perform the SUMO1 conjugation of Drp1. The addition of purified SENP2 efficiently was able to reverse this sumoylation process (a ∼3.8-fold decrease), suggesting Drp1 as a direct substrate of SENP2 (Figure 4C). Because of differential subcellular distributions of the SENP2 isoforms (SENP2 in nucleus; SENP2M in Golgi; SENP2S in cytoplasm) [16], their co-localizations with Drp1 were then investigated. Double labeling analysis indicated an extensive co-localization between Drp1 and SENP2S (Figure 4D). Using a proximity ligation assay examining protein-protein association within the cells, we found that SENP2S exhibited an isoform-specific interaction with Drp1 (Figure 4E). The interaction apparently took place in the mitochondria and cytoplasm (Figure 4F). Furthermore, using siRNA specifically knocking down SENP2 to an expression level at ∼17% (Figure 4H), we found that its reduction promotes Drp1 association with the mitochondria (Figure 4G), resulting in a 2.2-fold increase compared to the control (Figure 4H). A mitochondrial protein with higher molecular mass, which is probably the SUMO1-associated Drp1, was also increased in the SENP2 siRNA treated cells. Consistent with our analysis in the primary neuron (Figure 2C), the knockdown of SENP2 also enhanced mitochondrial fragmentation in the cell line (Figure 4I, p<0.01, ∼200 cells were counted in each of 3 independent experiments, mean ± SEM). These results imply an isoform-specific effect of SENP2 on Drp1 stabilization and mitochondrial accumulation through modulation of SUMO1-specific conjugation.

**Figure 5:**
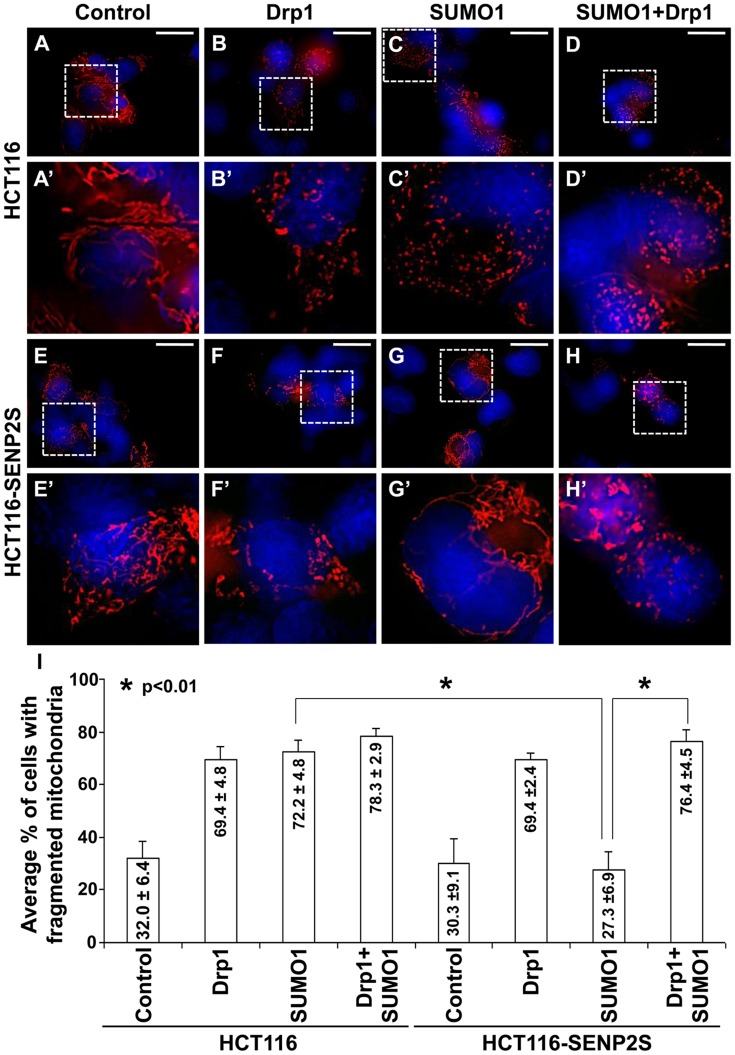
SENP2-dependent regulation of mitochondrial dynamics is mediated through modulation of Drp1. Mitochondrial morphology examined by DsRed2-Mito labeling (A–H, A′–H′) reveals that overexpression of Drp1 induces mitochondrial fission in HCT116 (A–B, A′–B′) and HCT116-SENP2S (E–F, E′–F′). SUMO1 mediated mitochondrial fragmentation in HCT116 cells (A, A′, C, C′) is prohibited in HCT116-SENP2S (E, E′, G, G′) cells. High levels of Drp1 are capable of reversing the protected effect of SENP2 on mitochondrial fission (D, D′, H, H′). Enlargement of the insets (A–H) are shown in A′–H′. (I) Graphs show the statistical analysis of mitochondrial morphology affected by Drp1 and SENP2 (p<0.01, n = 3, mean ± SEM). Scale bars, 20 µm (A–H).

The isoform-specific regulation of Drp1 by SENP2S suggests its potential involvement in modulating mitochondrial dynamics. Using DsRed2-Mito labeling, mitochondrial morphology was examined in HCT116 and HCT116-SENP2S cells. Similar to previous findings [28], overexpression of Drp1 and SUMO1 caused fragmentation of the mitochondria in these cells (Figure 5A–B, A′–B′, I). However, the SUMO1-induced mitochondrial fission was prohibited by high levels of SENP2 (Figure 5C, C′, G, G′, I, p<0.01, ∼100 cells were counted in each of 3 independent experiments, mean ± SEM). This might be attributed to the regulatory effects of SENP2 on Drp1 sumoylation and stability. Therefore, we examined if Drp1 is involved in the SENP2-mediated protection of mitochondrial fragmentation. High levels of Drp1 were able to overcome the protective effect of SENP2 on the SUMO1-induced mitochondrial fission (Figure 5D, D′, H, H′, I, p<0.01, ∼300 cells counted, n = 3, mean ± SEM). In contrast, high levels of SENP2S did not seem to affect the Drp1-induced mitochondrial fission, suggesting that Drp1 acts downstream of SENP2S in the regulatory pathway. These results not only indicated a role of SENP2 in controlling mitochondrial dynamics but also suggested that SENP2 exerts its effects through modulation of Drp1.

## Discussion

This study demonstrates that SENP2 controls the SUMO1-mediated modification of Drp1 essential for the regulation of mitochondrial dynamics. Targeted disruption of SENP2 induces neurodegeneration through promotion of Drp1 sumoylation and mitochondrial fragmentation. Impaired desumoylation results in neural cell death suggesting a new pathogenic mechanism for neurodegenerative diseases. Dysregulation of several aggregation-prone proteins which are sumoylation substrates have been implicated in neurodegeneration [19], [20], [22], [29], [30]. However, there is no evidence showing a direct involvement of SUMO modification enzymes in human diseases. Our findings suggest that enhanced sumoylation may also be attributed to mutations in the SUMO regulators in addition to the substrates. A balanced sumoylation is pivotal for neuronal cell survival. Hyper-sumoylation resulting from stimulation of SUMO ligases or disruption of SUMO proteases can lead to neural cell death. Our findings imply that targeting the SUMO protease may correct an imbalance of sumoylation and desumoylation. The SENP2^ΔSUMO-Nes^ mice might have potential in modeling human diseases associated with the SUMO pathway.

An association of the SUMO pathway with the regulation of mitochondrial dynamics has also been demonstrated in this study. Mitochondrial dysfunction has a strong association with neurodegenerative diseases [31]–[33]. Mitochondria possess a highly dynamic nature, undergoing frequent fusion and fission [34]. Due to large energy demands and long extended processes of the neurons, they are particularly sensitive and vulnerable to mitochondrial abnormalities. Enhanced mitochondrial fission induces apoptosis during neurodegeneration [31]–[33]. Mitochondrial dynamics is regulated by the GTPase dynamin-related protein Drp1, whose function is modulated by SUMO modification. In cells, overexpression of SUMO1 prevents Drp1 degradation, resulting in its stabilization and activation [35]. The SUMO1-induced Drp1 promotes mitochondrial fission which can be altered by manipulating the SENP activity [36], [37]. Data presented in this study strongly suggest that SENP2 is the physiological enzyme essential for this regulation. SENP2 controls mitochondrial dynamics through modulation of Drp1 in neural development and disease. Furthermore, Drp1 regulation by the SUMO pathway is causally linked to neural degeneration.

The SENP2 deficiency causes cell survival issues through increases in mitochondrial fission, leading to the development of neurodegeneration. As Drp1 appears to be a direct substrate of SENP2, dysregulation of mitochondrial dynamics is likely the primary cause of defects induced by the SENP2 disruption. Further study of mice with aberrant expression of Drp1 in the neural cells promises new insight into this regulatory mechanism. It remains possible that the aberrant mitochondrial phenotype is one of the main causes, which acts parallel with another cellular abnormality or is a consequence of other cellular abnormalities, e.g. failure of neural connection. Therefore, it is interesting to test if prevention of mitochondrial apoptosis can alleviate the defects caused by SENP2 deficiency. Further examination on the role of mitochondria dynamics promises new insight into the SENP2-mediated neuronal cell death.

The involvement of SENP2 in neural development and degeneration opens new opportunities to develop therapeutic targets in the SUMO pathway. As sumoylation has been shown to counter against ubiquitination, manipulation of the SUMO pathway may also alter the ubiquitination-mediated degradation for the prevention and treatment of neurological disorders. Although SENP2 may have a general effect on the neurons, it remains possible that a specific subtype is more sensitive to the loss of SENP2. In the SENP2 mutants, we identify different degrees of neurodegeneration in the cerebral cortex, hippocampus, cerebellum and spinal cord with the cerebral cortex being most severely affected. A disruption of SENP2 in a specific neuronal subtype may further divulge its role in neurodegenerative diseases. Testing the protective role of SENP2 in neural cell survival in disease conditions is also likely to gain a knowledge base of neurodegenerative diseases, leading to new therapeutic strategies.

## Materials and Methods

### DNA and cell

The pCS2-SENP2, pHASUMO1, pGEX-4T-SAE1/2, pGEX-2T-Ubc9, pCS2-SENP2, pCS2-SENP2M and pCS2-SENP2S DNA plasmids were described previously [16]. The pGEX-2T-Drp1 clone was generated by inserting a DNA fragment encoding *Drp1* into the pGEX-2T vector (GE HealthCare). The SRa-HA-SUMO2, pcDNA3-HA-SUMO3 and pDsRed2-Mito clones were from Addgene or Clontech Laboratories. C3H10T1/2 and HCT116 cells and their derivatives were cultured in DMEM media with 10% fetal bovine serum and antibiotics [16], [38]. Isolation, culture and differentiation of primary neural progenitor cells were performed as described [38], [39].

### Mouse strains

The SENP2^ΔSUMO^Fx ES cell lines were generated by electroporation of a targeting vector, containing the insertion of an orphan loxP site in intron 15 and another loxP site and a pgk-neo cassette flanked by two FRT sites in intron 16, into CSL3 ES cells [17], [40], [41]. Twenty mouse ES cell clones heterozygous for the targeted allele were obtained by homologous recombination (targeting efficiency: 23/112). Two independent clones were injected into blastocysts to generate chimeras which were bred to obtain mice carrying the targeted allele. These mice were then crossed with the R26Flp mice to remove the pgk-neo cassette to obtain the SENP2^ΔSUMO^Fx mouse strain. Mice were genotyped by PCR analysis using primers (5′-TCTCACTTGAAACCGTAGGGACC-3′ and 5′-GAAGGAAGGACTGGAGGAGAGAAG-3′) to identify the 5′ loxP locus, primers (5′-TTGTCAGAAGCAGTGTCCTGCG-3′ and 5′-GACTGGGAAGATATGAACTCGGC-3′) to identify the 3′ loxP locus. The deleted allele was identified using primers (5′-TCTCACTTGAAACCGTAGGGACC-3′ and 5′-GACTGGGAAGATATGAACTCGGC-3′). The PCR was performed by denaturation at 95°C for 5 min and 35 cycles of amplification (95°C for 30 s, 67°C for 30 s, and 72°C for 60 s), followed by a 7-min extention at 72°C. The SENP2^lacZ^ and Nestin-Cre mouse strains and genotyping methods were reported previously [17], [40]. To generate the *SENP2^ΔSUMO^* mouse strain expressing a deficient protein, mice carrying the *SENP2^ΔSUMO^Fx* allele were crossed with EIIa-Cre transgenic mice to delete the protease core domain in the germ cells [41]. To examine the production of SENP2 transcript, the reverse transcription products were subject to PCR amplifications using primers 5′-CAGTCTCTACAATGCTGCC-3′ and 5′-CTGTCACTCTGATCTTTGG-3′ (exons 3–5), primers 5′-GTGAGCTCATGAGTTCTGG-3′ and 5′-GTCGCTCCAATAACTTTCG-3′ (exons 5–7), primers 5′-GGAGGAGCAGAATCATGG-3′ and 5′-CTCAAAATCTCATCTGGTGG-3′(exons 8–11) and primers 5′-AGGTACATTGGAGCCTGGTG-3′ and 5′-AGCAACTGCTGGTGAAGGAT-3′ (exons 13–17). The PCR reaction was performed by denaturation at 94°C for 5 min and 30 cycles of amplification (94°C for 30 s, 53°C for 30 s, and 72°C for 45 s), followed by a 7-min extension at 72°C. Care and use of experimental animals described in this work were approved by and comply with guidelines and policies of the University of Committee on Animal Resources at the University of Rochester.

### Histology, in situ hybridization and TUNEL analysis

Samples were fixed, paraffin embedded, sectioned and stained with hematoxylin/eosin for histological evaluation [17],[42]. The in situ hybridization was performed as described [17], [38], [43], [44]. In brief, sections were incubated with the digoxygenin labeled RNA probes generated by in vitro transcription [17], [43], followed by recognition with an alkaline phosphatase conjugated anti-digoxygenin antibody and visualization with BM-purple [38], [44]. TUNEL staining was performed with ApopTag (Millipore) as described [45], [46]. For electron microscopy, mice were fixed by perfusion with fixative (2% paraformaldehyde, 2.5% Glutaraldehyde, 0.1M sodium cacodylate, 6.8% sucrose). The dissected tissues were then fixed with 1% osmium tetroxide, embedded in EPON/Araldite resin and cut in seventy nm sections, followed by staining with aqueous uranyl acetate and lead citrate and examined using Hitachi 7650 transmission electron microscope.

### Proximity ligation assay, immunostaining, immunoblot and immunoprecipitation

Proximity ligation assay (PLA) was performed using Duolink In Situ reagents (Duolink Bioscience). Briefly, cells were fixed and incubated with rabbit anti-Drp1 and mouse anti-myc tag antibodies. Two oligonucleotide-labeled anti-rabbit and anti-mouse PLA probes, which bind to each other when they are in close proximity, were then used to generate fluorescent signals. Immunostaining of cells [47] and tissue sections [48]–[50] were performed by incubation with primary antibodies, followed by detection with fluorescence-conjugated or horseradish peroxidase-conjugated secondary antibodies. Images were taken using Zeiss Axio Observer microscope equipped with deconvolution analysis. To determine the mitochondrial morphology, cells were either stained by MitoTracker or transient expression of DsRed2-Mito. For statistical analysis, cells containing MitoTracker or DsRed2-Mito positive mitochondria were counted and scored for tubular/rod-like or fragmented mitochondria. Immunoblot was performed by isolation of protein extracts from mitochondria, cells or tissues using M-PER (Pierce) in the presence of protease inhibitor cocktail, followed by electrophoresis as described [16], [17], [38], [48]. Isolation of mitochondria was performed using a Mitochondria Isolation Kit (Thermo Fisher) according to the manufacture's description. Immunoprecipitation was performed using Pierce Classic IP Kit. Briefly, cells were lysed in buffer containing 0.025M Tris, 0.15M NaCl, 0.001M EDTA, 1% NP-40, 5% glycerol. Approximately 500 µg of protein lysates were mixed with 2 µg of antibodies overnight, followed by incubation with Protein A/G agarose for 1 hour at 4°C. The antibody-bound complex was then incubated with elution buffer for 5 min at 100°C, and collected by centrifugation for SDS-PAGE analysis. Mouse monoclonal antibodies, Actin (Thermo Fisher), HA (Cell Signaling), and myc tag (Santa Cruz); rabbit polyclonal antibodies Bak (Novus Biologicals), caspase-3 (BD Biosciences), Cox IV (Cell Signaling), Drp1 (Novus Biologicals), and SUMO1 (Cell Signaling), were used in these analyses.

### Sumoylation and desumoylation

Recombinant GST-SAE1/SAE2, GST-Ubc9, GST-Drp1, HA-SUMO1 and myc tagged (MT)-SENP2 proteins expressed in *Escherichia coli* were affinity purified. The 20-µl reaction buffer containing 50 mM Tris-HCl (pH 7.6), 5 mM magnesium chloride, 10 mM ATP, 1 µg of GST-ASE1/2, 2 µg of GST-Ubc9, 10 µg of GST-HA-SUMO1 and 200 ng of GST-Drp1 with the presence of protease inhibitor cocktail was incubated for 3 h at 37°C. The desumoylation reaction was then carried out in 10 µl of the above sumoylation mixture with the addition of purified MT-SENP2 for overnight at 37°C. The samples were then analyzed by SDS-PAGE and immunoblot analysis of Drp1 and SUMO1.

## Supporting Information

Figure S1 Whole mount (A, B) and histological (C, D) analyses of the E10.5 SENP2+/+ (A, C) and SENP2−/− (B, D) embryos reveal brain abnormalities (arrows) caused by total knockout (embryonic and extra-embryonic ablation) of SENP2. TUNEL staining in whole mounts (E, F) and sections (G, H) identifies an increase in apoptotic cells associated with the SENP2 deletion. The images shown are representatives of more than three independent experiments. Scale bars, 1 mm (A–F); 100 µm (G, H).
(TIF)

Figure S2 Diagram illustrates the creation of mice carrying SENP2^ΔSUMO^Fx allele. (A) Exon 16 (Ex16), containing the protease core domain, is flanked by two loxP sites. Removal of exon 16 causes an in-frame deletion, resulting in production of an internally truncated SENP2 protein. (B) Southern blot analysis with 3′ and 5′ external probes identifies ES cell clones carrying the targeted allele. (C) PCR analysis detects the presence of 5′ (P1–P2) and 3′ (P3–P4) loxP sites for genotyping the wild-type (+/+) and heterozygous (Fx/+) SENP2^ΔSUMO^Fx mice, and examines the deletion of exon 16 in the SENP2^ΔSUMO^Δ/+ mice (P1–P4). (D) RT-PCR analyzes the SENP2 RNA transcribed in the wild type (+/+), heterozygous (+/−) and homozygous (−/−) embryos. No difference in exon 3–5, exon 5–7 and exon 8–11 is found. A smaller RT-PCR product for exon 13–17 is detected in the mutant due to Cre-mediated in-frame deletion of exon 16.
(TIF)

Figure S3 The SENP2^ΔSUMO^ homozygous mutants exhibit embryonic and extraembryonic abnormalities. Whole mount (A–B) and histological (C–J) evaluations of the E10.5 wild type (A, C, E, G, I) and mutant (B, D, F, H, J) embryos (A–B) and placentas (C–J) reveal that deletion of the SUMO protease core domain results in embryonic and extraembryonic defects highly reminiscent to the SENP2 nulls. Labyrinth (L), spongiotrophoblast (S), trophoblast giant cell (G) layers are defined by blue, red and green broken lines, respectively. Arrow and arrowheads indicate maternal and fetal blood spaces, respectively. The images shown are representatives of three independent experiments. Scale bars, 1 mm (A–B), 500 µm (C–D), 50 µm (E–J).
(TIF)

Figure S4 Nestin-Cre transgene induces site-specific recombination in neural development. The efficacy of DNA recombination medicated by Nestin-Cre is examined by an R26RlacZ reporter allele. Examination of embryos carrying Nestin-Cre and R26RlacZ by β-gal staining in whole mount (A–B, C–D) and section (a–d) demonstrates the efficacy of Cre-mediated recombination in neural progenitor cells at E10.5 (A–B, a–c) and E12.5 (C–D, d). The approximate positions of a–d are shown by the broken line in B and D. fb, forebrain; hb, hindbrain; mb, midbrain; nt, neural tube. Scale bars, 1 mm (A–D, d); 100 µm (a–c).
(TIF)

Figure S5 The loss of SENP2 affects CNS development. Hematoxylin and eosin staining analyzes development of the control (genotype: Nestin-Cre; SENP2^SUMO^Fx/+ and SENP2^SUMO^Fx/Fx) and SENP2^ΔSUMO-Nes^ midbrains, cerebella, hippocampi and spinal cords at P14. The mutation causes size reduction in these areas. Compared to the control, mutant neural cells are loosely distributed (arrows), presenting evidence for neurodegeneration. Enlargements of the insets (A–H) are shown in A′–H′. Scale bars, 500 µm (A–H); 50 µm (A′–H′).
(TIF)

Figure S6 Abnormal apoptosis is detected in the SENP2 mutants during CNS development. TUNEL staining examines apoptotic cells in the control (genotype: Nestin-Cre; SENP2^SUMO^Fx/+ and SENP2^SUMO^Fx/Fx) and SENP2^ΔSUMO-Nes^ midbrains, cerebella, hippocampi and spinal cords at P7. The mutation enhances apoptosis in these areas. Enlargements of the insets (A–H) are shown in A′–H′. The images shown are representatives of three independent experiments. Scale bars, 500 µm (A–H); 50 µm (A′–H′).
(TIF)

Figure S7 The deletion of SENP2 affects Drp1 association with the mitochondria. Co-labeling of mitochondria by MitoTracker (red), endogenous Drp1 by immunostaining (green), and nuclei (blue), shows differential association of Drp1 with the mitochondria in control and mutant. Enlargements of the inset are shown in the bottom panel. Arrows, arrowheads and asterisk indicate tubular/rod-like mitochondria, fragmented mitochondria and Drp1 association with the mitochondria, respectively. The images are representatives of three independent experiments. Scale bars, 20 µm.
(TIF)

Figure S8 SUMO2 and SUMO3 are not involved in the SENP2-mediated modification of Drp1. (A) Immunoblot (IB) analysis examines the expression of HA-SUMO2, Drp1 and Actin in HCT116 cells and in HCT116 stably transformed variants, HCT116-SENP2, HCT116-SENP2M and HCT116-SENP2S. (B) IB analysis examines the expression of HA-SUMO3, Drp1 and Actin in HCT116 cells and in HCT116 stably transformed variants, HCT116-SENP2, HCT116-SENP2M and HCT116-SENP2S. Overexpression of HA-SUMO2 (A) or HA-SUMO3 (B) induces sumoylation in HCT116 cells and in HCT116 stably transformed variants, HCT116-SENP2, HCT116-SENP2M and HCT116-SENP2S. No significant difference in Drp1 expression is found in HCT116 cells and HCT116 variants. Actin level serves as a protein loading control.
(TIF)

Video S1 A clip shows movement difficulties of the P10 SENP2^ΔSUMO-Nes^ mice which further developed paralysis around P16.
(MOV)

## References

[1] Martin S, Nishimune A, Mellor JR, Henley JM (2007) SUMOylation regulates kainate-receptor-mediated synaptic transmission. Nature 447: 321–325. 17486098 10.1038/nature05736 PMC3310901

[2] Shalizi A, Gaudilliere B, Yuan Z, Stegmuller J, Shirogane T, et al (2006) A calcium-regulated MEF2 sumoylation switch controls postsynaptic differentiation. Science 311: 1012–1017. 16484498 10.1126/science.1122513

[3] Flavell SW, Cowan CW, Kim TK, Greer PL, Lin Y, et al (2006) Activity-dependent regulation of MEF2 transcription factors suppresses excitatory synapse number. Science 311: 1008–1012. 16484497 10.1126/science.1122511

[4] Krumova P, Weishaupt JH (2013) Sumoylation in neurodegenerative diseases. Cell Mol Life Sci 70: 2123–2138. 23007842 10.1007/s00018-012-1158-3 PMC11113377

[5] Lieberman AP (2004) SUMO, a ubiquitin-like modifier implicated in neurodegeneration. Exp Neurol 185: 204–207. 14736501 10.1016/j.expneurol.2003.10.009

[6] Martin S, Wilkinson KA, Nishimune A, Henley JM (2007) Emerging extranuclear roles of protein SUMOylation in neuronal function and dysfunction. Nat Rev Neurosci 8: 948–959. 17987030 10.1038/nrn2276 PMC3314512

[7] Seeler JS, Dejean A (2003) Nuclear and unclear functions of SUMO. Nat Rev Mol Cell Biol 4: 690–699. 14506472 10.1038/nrm1200

[8] Ouyang J, Valin A, Gill G (2009) Regulation of transcription factor activity by SUMO modification. Methods Mol Biol 497: 141–152. 19107415 10.1007/978-1-59745-566-4_9

[9] Geoffroy MC, Hay RT (2009) An additional role for SUMO in ubiquitin-mediated proteolysis. Nat Rev Mol Cell Biol 10: 564–568. 19474794 10.1038/nrm2707

[10] Cubenas-Potts C, Matunis MJ (2013) SUMO: a multifaceted modifier of chromatin structure and function. Dev Cell 24: 1–12. 23328396 10.1016/j.devcel.2012.11.020 PMC3555686

[11] Ulrich HD (2009) The SUMO system: an overview. Methods Mol Biol 497: 3–16. 19107407 10.1007/978-1-59745-566-4_1

[12] Melchior F (2000) SUMO–nonclassical ubiquitin. Annu Rev Cell Dev Biol 16: 591–626. 11031248 10.1146/annurev.cellbio.16.1.591

[13] Gareau JR, Lima CD (2010) The SUMO pathway: emerging mechanisms that shape specificity, conjugation and recognition. Nat Rev Mol Cell Biol 11: 861–871. 21102611 10.1038/nrm3011 PMC3079294

[14] Melchior F, Schergaut M, Pichler A (2003) SUMO: ligases, isopeptidases and nuclear pores. Trends Biochem Sci 28: 612–618. 14607092 10.1016/j.tibs.2003.09.002

[15] Hickey CM, Wilson NR, Hochstrasser M (2012) Function and regulation of SUMO proteases. Nat Rev Mol Cell Biol 13: 755–766. 23175280 10.1038/nrm3478 PMC3668692

[16] Jiang M, Chiu SY, Hsu W (2011) SUMO-specific protease 2 in Mdm2-mediated regulation of p53. Cell Death Differ 18: 1005–1015. 21183956 10.1038/cdd.2010.168 PMC3081924

[17] Chiu SY, Asai N, Costantini F, Hsu W (2008) SUMO-Specific Protease 2 Is Essential for Modulating p53-Mdm2 in Development of Trophoblast Stem Cell Niches and Lineages. PLoS Biol 6: e310. 19090619 10.1371/journal.pbio.0060310 PMC2602722

[18] Kang X, Qi Y, Zuo Y, Wang Q, Zou Y, et al (2010) SUMO-specific protease 2 is essential for suppression of polycomb group protein-mediated gene silencing during embryonic development. Mol Cell 38: 191–201. 20417598 10.1016/j.molcel.2010.03.005 PMC2879644

[19] Shinbo Y, Niki T, Taira T, Ooe H, Takahashi-Niki K, et al (2006) Proper SUMO-1 conjugation is essential to DJ-1 to exert its full activities. Cell Death Differ 13: 96–108. 15976810 10.1038/sj.cdd.4401704

[20] Steffan JS, Agrawal N, Pallos J, Rockabrand E, Trotman LC, et al (2004) SUMO modification of Huntingtin and Huntington's disease pathology. Science 304: 100–104. 15064418 10.1126/science.1092194

[21] Pountney DL, Huang Y, Burns RJ, Haan E, Thompson PD, et al (2003) SUMO-1 marks the nuclear inclusions in familial neuronal intranuclear inclusion disease. Exp Neurol 184: 436–446. 14637113 10.1016/j.expneurol.2003.07.004

[22] Riley BE, Zoghbi HY, Orr HT (2005) SUMOylation of the polyglutamine repeat protein, ataxin-1, is dependent on a functional nuclear localization signal. J Biol Chem 280: 21942–21948. 15824120 10.1074/jbc.M501677200

[23] Hattori N, Mizuno Y (2004) Pathogenetic mechanisms of parkin in Parkinson's disease. Lancet 364: 722–724. 15325839 10.1016/S0140-6736(04)16901-8

[24] Ren D, Tu HC, Kim H, Wang GX, Bean GR, et al (2010) BID, BIM, and PUMA are essential for activation of the BAX- and BAK-dependent cell death program. Science 330: 1390–1393. 21127253 10.1126/science.1190217 PMC3163443

[25] Wasiak S, Zunino R, McBride HM (2007) Bax/Bak promote sumoylation of DRP1 and its stable association with mitochondria during apoptotic cell death. J Cell Biol 177: 439–450. 17470634 10.1083/jcb.200610042 PMC2064824

[26] Cho DH, Nakamura T, Fang J, Cieplak P, Godzik A, et al (2009) S-nitrosylation of Drp1 mediates beta-amyloid-related mitochondrial fission and neuronal injury. Science 324: 102–105. 19342591 10.1126/science.1171091 PMC2823371

[27] Kim J, Moody JP, Edgerly CK, Bordiuk OL, Cormier K, et al (2010) Mitochondrial loss, dysfunction and altered dynamics in Huntington's disease. Hum Mol Genet 19: 3919–3935. 20660112 10.1093/hmg/ddq306 PMC2947400

[28] Pitts KR, Yoon Y, Krueger EW, McNiven MA (1999) The dynamin-like protein DLP1 is essential for normal distribution and morphology of the endoplasmic reticulum and mitochondria in mammalian cells. Mol Biol Cell 10: 4403–4417. 10588666 10.1091/mbc.10.12.4403 PMC25766

[29] Krumova P, Meulmeester E, Garrido M, Tirard M, Hsiao HH, et al (2011) Sumoylation inhibits alpha-synuclein aggregation and toxicity. J Cell Biol 194: 49–60. 21746851 10.1083/jcb.201010117 PMC3135405

[30] Li Y, Wang H, Wang S, Quon D, Liu YW, et al (2003) Positive and negative regulation of APP amyloidogenesis by sumoylation. Proc Natl Acad Sci U S A 100: 259–264. 12506199 10.1073/pnas.0235361100 PMC140945

[31] Bender T, Martinou JC (2013) Where killers meet–permeabilization of the outer mitochondrial membrane during apoptosis. Cold Spring Harb Perspect Biol 5: a011106. 23284044 10.1101/cshperspect.a011106 PMC3579396

[32] Mali P, Esvelt KM, Church GM (2013) Cas9 as a versatile tool for engineering biology. Nat Methods 10: 957–963. 24076990 10.1038/nmeth.2649 PMC4051438

[33] Reddy PH, Reddy TP, Manczak M, Calkins MJ, Shirendeb U, et al (2011) Dynamin-related protein 1 and mitochondrial fragmentation in neurodegenerative diseases. Brain Res Rev 67: 103–118. 21145355 10.1016/j.brainresrev.2010.11.004 PMC3061980

[34] Chan DC (2012) Fusion and fission: interlinked processes critical for mitochondrial health. Annu Rev Genet 46: 265–287. 22934639 10.1146/annurev-genet-110410-132529

[35] Harder Z, Zunino R, McBride H (2004) Sumo1 conjugates mitochondrial substrates and participates in mitochondrial fission. Curr Biol 14: 340–345. 14972687 10.1016/j.cub.2004.02.004

[36] Zunino R, Schauss A, Rippstein P, Andrade-Navarro M, McBride HM (2007) The SUMO protease SENP5 is required to maintain mitochondrial morphology and function. J Cell Sci 120: 1178–1188. 17341580 10.1242/jcs.03418

[37] Zunino R, Braschi E, Xu L, McBride HM (2009) Translocation of SenP5 from the nucleoli to the mitochondria modulates DRP1-dependent fission during mitosis. J Biol Chem 284: 17783–17795. 19411255 10.1074/jbc.M901902200 PMC2719417

[38] Fu J, Jiang M, Mirando AJ, Yu HM, Hsu W (2009) Reciprocal regulation of Wnt and Gpr177/mouse Wntless is required for embryonic axis formation. Proc Natl Acad Sci U S A 106: 18598–18603. 19841259 10.1073/pnas.0904894106 PMC2773984

[39] Yu HM, Jin Y, Fu J, Hsu W (2010) Expression of Gpr177, a Wnt trafficking regulator, in mouse embryogenesis. Dev Dyn 239: 2102–2109. 20549736 10.1002/dvdy.22336 PMC2894299

[40] Yu HM, Liu B, Chiu SY, Costantini F, Hsu W (2005) Development of a unique system for spatiotemporal and lineage-specific gene expression in mice. Proc Natl Acad Sci U S A 102: 8615–8620. 15941831 10.1073/pnas.0500124102 PMC1150815

[41] Fu J, Ivy Yu HM, Maruyama T, Mirando AJ, Hsu W (2011) Gpr177/mouse Wntless is essential for Wnt-mediated craniofacial and brain development. Dev Dyn 240: 365–371. 21246653 10.1002/dvdy.22541 PMC3056068

[42] Yu HM, Jerchow B, Sheu TJ, Liu B, Costantini F, et al (2005) The role of Axin2 in calvarial morphogenesis and craniosynostosis. Development 132: 1995–2005. 15790973 10.1242/dev.01786 PMC1828115

[43] Fu J, Hsu W (2013) Epidermal Wnt controls hair follicle induction by orchestrating dynamic signaling crosstalk between the epidermis and dermis. J Invest Dermatol 133: 890–898. 23190887 10.1038/jid.2012.407 PMC3594635

[44] Maruyama T, Jiang M, Hsu W (2013) Gpr177, a novel locus for bone mineral density and osteoporosis, regulates osteogenesis and chondrogenesis in skeletal development. J Bone Miner Res 28: 1150–1159. 23188710 10.1002/jbmr.1830 PMC3593783

[45] Maruyama T, Mirando AJ, Deng CX, Hsu W (2010) The balance of WNT and FGF signaling influences mesenchymal stem cell fate during skeletal development. Sci Signal 3: ra40. 20501936 10.1126/scisignal.2000727 PMC2902546

[46] Yu HM, Liu B, Costantini F, Hsu W (2007) Impaired neural development caused by inducible expression of Axin in transgenic mice. Mech Dev 124: 146–156. 17123792 10.1016/j.mod.2006.10.002 PMC1847614

[47] Liu B, Yu HM, Hsu W (2007) Craniosynostosis caused by Axin2 deficiency is mediated through distinct functions of beta-catenin in proliferation and differentiation. Dev Biol 301: 298–308. 17113065 10.1016/j.physletb.2003.10.071 PMC1821096

[48] Liu B, Yu HM, Huang J, Hsu W (2008) Co-opted JNK/SAPK signaling in Wnt/beta-catenin-induced tumorigenesis. Neoplasia 10: 1004–1013. 18714362 10.1593/neo.08548 PMC2517646

[49] Mirando AJ, Maruyama T, Fu J, Yu HM, Hsu W (2010) Beta-catenin/cyclin D1 mediated development of suture mesenchyme in calvarial morphogenesis. BMC Dev Biol 10: 116. 21108844 10.1186/1471-213X-10-116 PMC3001432

[50] Maruyama EO, Yu HM, Jiang M, Fu J, Hsu W (2013) Gpr177 deficiency impairs mammary development and prohibits Wnt-induced tumorigenesis. PLoS One 8: e56644. 23457599 10.1371/journal.pone.0056644 PMC3574013

