# Supplementary figures and images for: Disruption of SUMO-Specific Protease 2 Induces Mitochondria Mediated Neurodegeneration
Source: PLoS Genet. 2014 Oct 9;10(10):e1004579. doi: 10.1371/journal.pgen.1004579 (PMC4191884; doi:10.1371/journal.pgen.1004579)

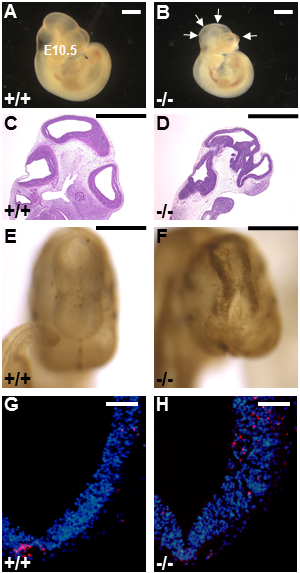

Supplement: Figure S1 — Whole mount (A, B) and histological (C, D) analyses of the E10.5 SENP2+/+ (A, C) and SENP2−/− (B, D) embryos reveal brain abnormalities (arrows) caused by total knockout (embryonic and extra-embryonic ablation) of SENP2. TUNEL staining in whole mounts (E, F) and sections (G, H) identifies an increase in apoptotic cells associated with the SENP2 deletion. The images shown are representatives of more than three independent experiments. Scale bars, 1 mm (A–F); 100 µm (G, H). (TIF) [file pgen.1004579.s001.tif]

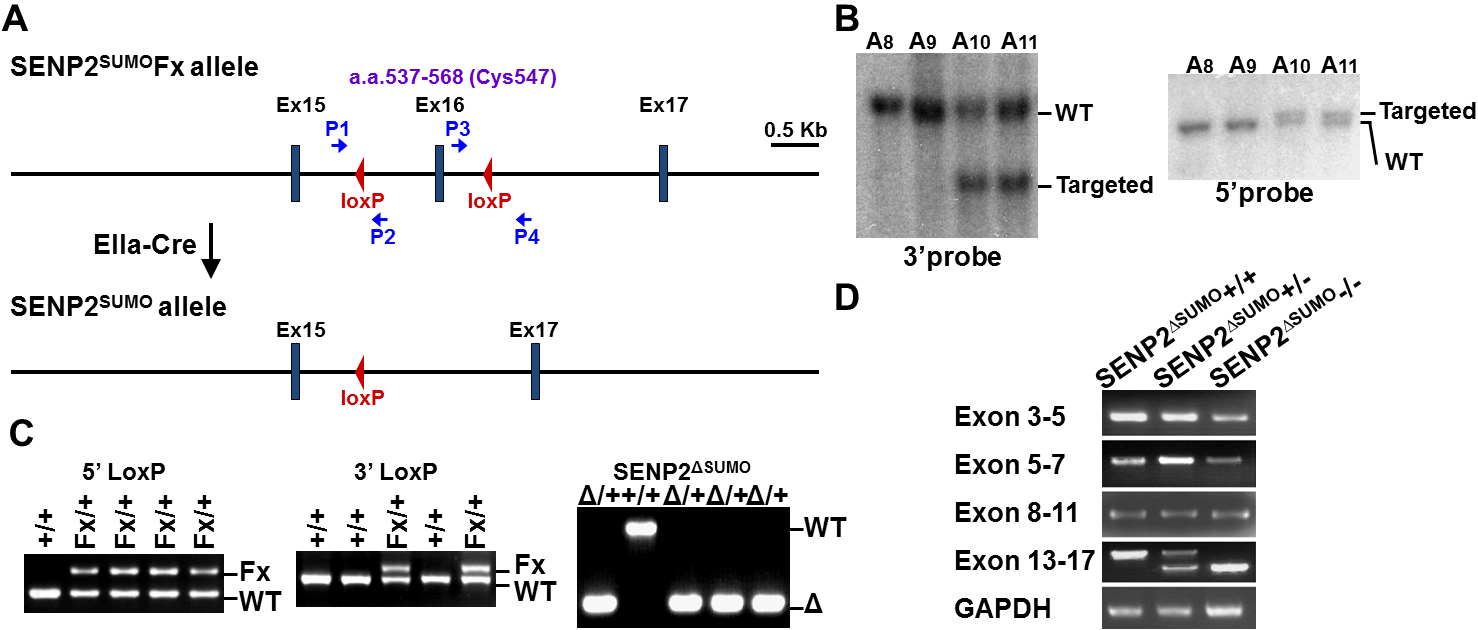

Supplement: Figure S2 — Diagram illustrates the creation of mice carrying SENP2ΔSUMOFx allele. (A) Exon 16 (Ex16), containing the protease core domain, is flanked by two loxP sites. Removal of exon 16 causes an in-frame deletion, resulting in production of an internally truncated SENP2 protein. (B) Southern blot analysis with 3′ and 5′ external probes identifies ES cell clones carrying the targeted allele. (C) PCR analysis detects the presence of 5′ (P1–P2) and 3′ (P3–P4) loxP sites for genotyping the wild-type (+/+) and heterozygous (Fx/+) SENP2ΔSUMOFx mice, and examines the deletion of exon 16 in the SENP2ΔSUMOΔ/+ mice (P1–P4). (D) RT-PCR analyzes the SENP2 RNA transcribed in the wild type (+/+), heterozygous (+/−) and homozygous (−/−) embryos. No difference in exon 3–5, exon 5–7 and exon 8–11 is found. A smaller RT-PCR product for exon 13–17 is detected in the mutant due to Cre-mediated in-frame deletion of exon 16. (TIF) [file pgen.1004579.s002.tif]

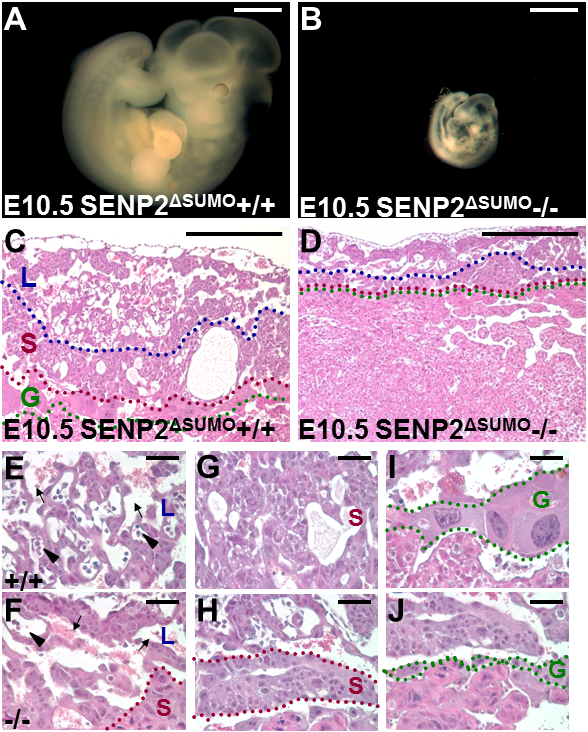

Supplement: Figure S3 — The SENP2ΔSUMO homozygous mutants exhibit embryonic and extraembryonic abnormalities. Whole mount (A–B) and histological (C–J) evaluations of the E10.5 wild type (A, C, E, G, I) and mutant (B, D, F, H, J) embryos (A–B) and placentas (C–J) reveal that deletion of the SUMO protease core domain results in embryonic and extraembryonic defects highly reminiscent to the SENP2 nulls. Labyrinth (L), spongiotrophoblast (S), trophoblast giant cell (G) layers are defined by blue, red and green broken lines, respectively. Arrow and arrowheads indicate maternal and fetal blood spaces, respectively. The images shown are representatives of three independent experiments. Scale bars, 1 mm (A–B), 500 µm (C–D), 50 µm (E–J). (TIF) [file pgen.1004579.s003.tif]

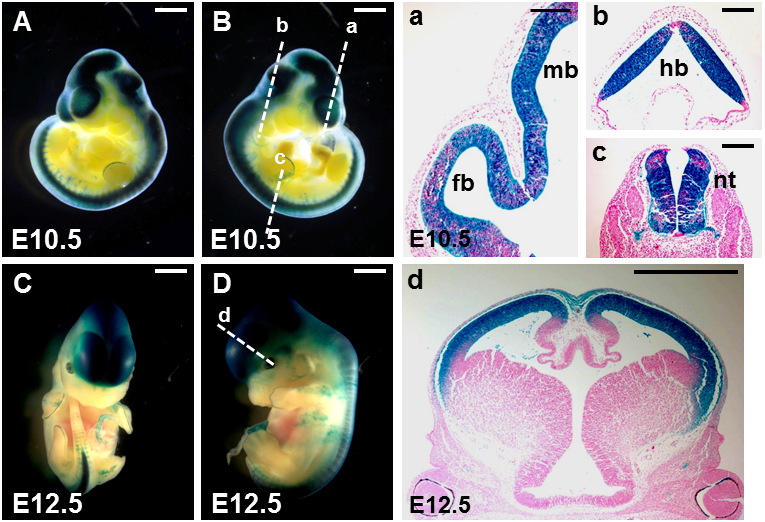

Supplement: Figure S4 — Nestin-Cre transgene induces site-specific recombination in neural development. The efficacy of DNA recombination medicated by Nestin-Cre is examined by an R26RlacZ reporter allele. Examination of embryos carrying Nestin-Cre and R26RlacZ by β-gal staining in whole mount (A–B, C–D) and section (a–d) demonstrates the efficacy of Cre-mediated recombination in neural progenitor cells at E10.5 (A–B, a–c) and E12.5 (C–D, d). The approximate positions of a–d are shown by the broken line in B and D. fb, forebrain; hb, hindbrain; mb, midbrain; nt, neural tube. Scale bars, 1 mm (A–D, d); 100 µm (a–c). (TIF) [file pgen.1004579.s004.tif]

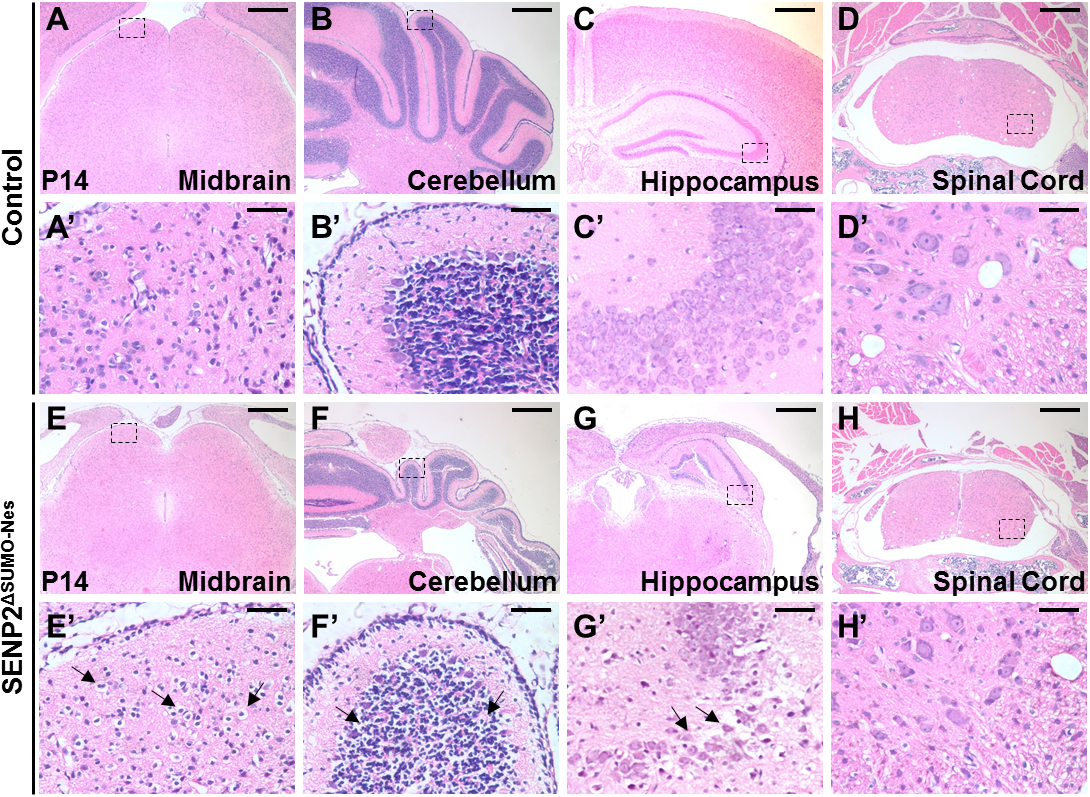

Supplement: Figure S5 — The loss of SENP2 affects CNS development. Hematoxylin and eosin staining analyzes development of the control (genotype: Nestin-Cre; SENP2SUMOFx/+ and SENP2SUMOFx/Fx) and SENP2ΔSUMO-Nes midbrains, cerebella, hippocampi and spinal cords at P14. The mutation causes size reduction in these areas. Compared to the control, mutant neural cells are loosely distributed (arrows), presenting evidence for neurodegeneration. Enlargements of the insets (A–H) are shown in A′–H′. Scale bars, 500 µm (A–H); 50 µm (A′–H′). (TIF) [file pgen.1004579.s005.tif]

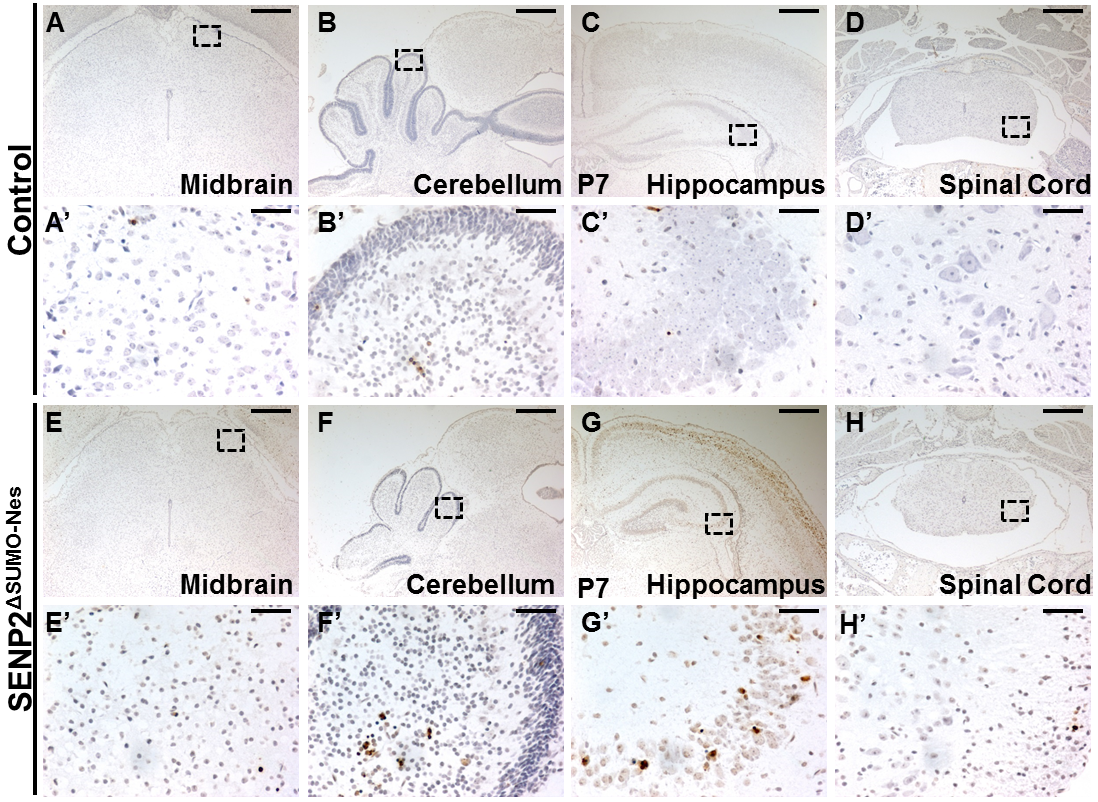

Supplement: Figure S6 — Abnormal apoptosis is detected in the SENP2 mutants during CNS development. TUNEL staining examines apoptotic cells in the control (genotype: Nestin-Cre; SENP2SUMOFx/+ and SENP2SUMOFx/Fx) and SENP2ΔSUMO-Nes midbrains, cerebella, hippocampi and spinal cords at P7. The mutation enhances apoptosis in these areas. Enlargements of the insets (A–H) are shown in A′–H′. The images shown are representatives of three independent experiments. Scale bars, 500 µm (A–H); 50 µm (A′–H′). (TIF) [file pgen.1004579.s006.tif]

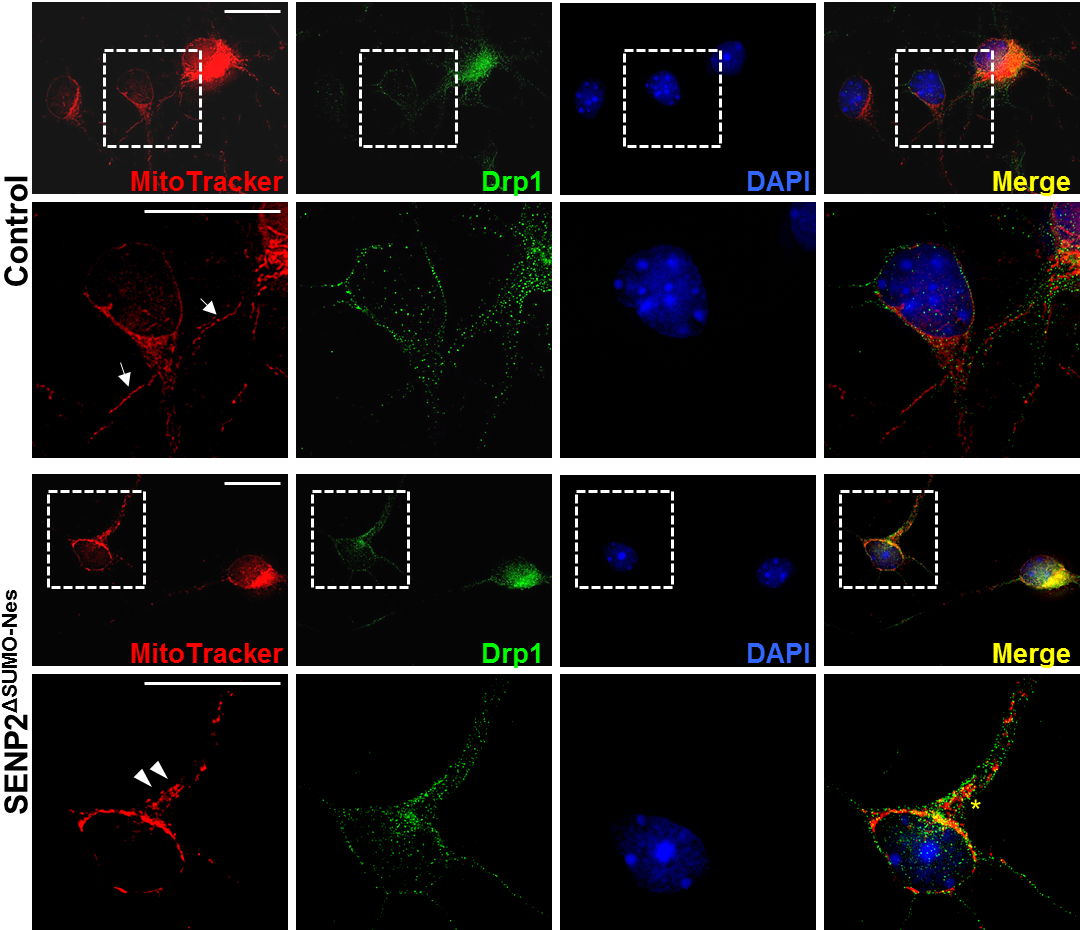

Supplement: Figure S7 — The deletion of SENP2 affects Drp1 association with the mitochondria. Co-labeling of mitochondria by MitoTracker (red), endogenous Drp1 by immunostaining (green), and nuclei (blue), shows differential association of Drp1 with the mitochondria in control and mutant. Enlargements of the inset are shown in the bottom panel. Arrows, arrowheads and asterisk indicate tubular/rod-like mitochondria, fragmented mitochondria and Drp1 association with the mitochondria, respectively. The images are representatives of three independent experiments. Scale bars, 20 µm. (TIF) [file pgen.1004579.s007.tif]

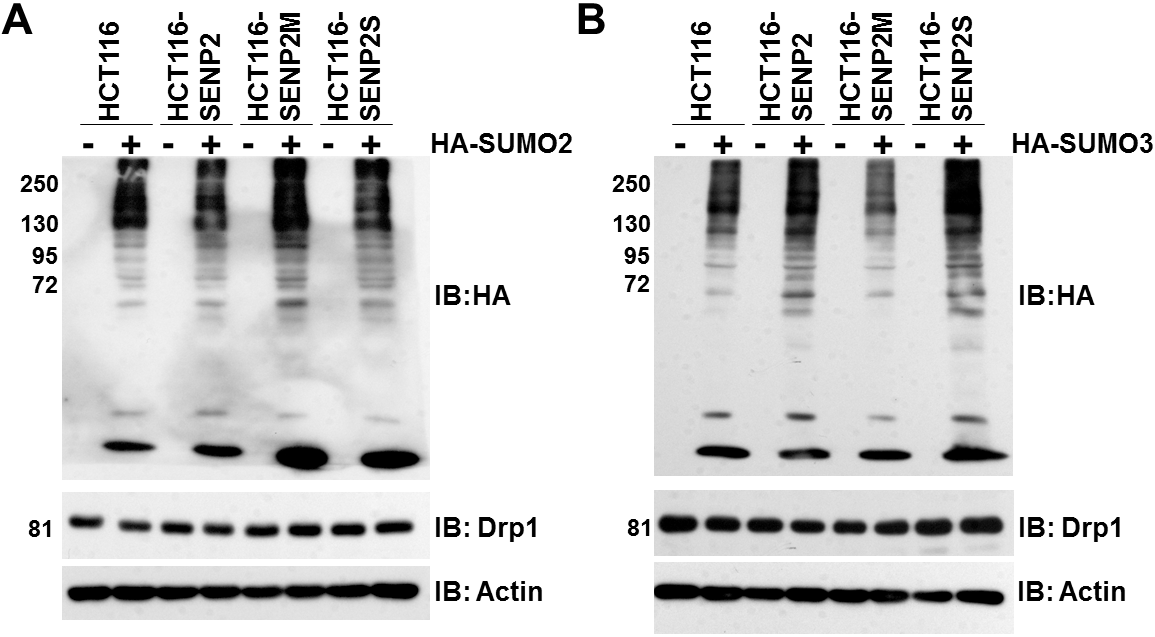

Supplement: Figure S8 — SUMO2 and SUMO3 are not involved in the SENP2-mediated modification of Drp1. (A) Immunoblot (IB) analysis examines the expression of HA-SUMO2, Drp1 and Actin in HCT116 cells and in HCT116 stably transformed variants, HCT116-SENP2, HCT116-SENP2M and HCT116-SENP2S. (B) IB analysis examines the expression of HA-SUMO3, Drp1 and Actin in HCT116 cells and in HCT116 stably transformed variants, HCT116-SENP2, HCT116-SENP2M and HCT116-SENP2S. Overexpression of HA-SUMO2 (A) or HA-SUMO3 (B) induces sumoylation in HCT116 cells and in HCT116 stably transformed variants, HCT116-SENP2, HCT116-SENP2M and HCT116-SENP2S. No significant difference in Drp1 expression is found in HCT116 cells and HCT116 variants. Actin level serves as a protein loading control. (TIF) [file pgen.1004579.s008.tif]
